# Aortic valve function post-replacement of severe aortic stenosis by transcatheter procedure versus surgery: a systematic review and metanalysis

**DOI:** 10.1038/s41598-021-91548-x

**Published:** 2021-06-07

**Authors:** Charbel Abi Khalil, Barbara Ignatiuk, Guliz Erdem, Hiam Chemaitelly, Fabio Barilli, Mohamed El-Shazly, Jassim Al Suwaidi, Samar Aboulsoud, Markus Kofler, Lukas Stastny, Hani Jneid, Nikolaos Bonaros

**Affiliations:** 1 Research Department, Weill Cornell Medicine-Qatar, Doha, Qatar; 2 Joan and Sanford I. Weill Department of Medicine, Weill Cornell Medicine, New York, USA; 3 Department of Health Policy, London School of Economics, London, UK; 4 Ospedali Riuniti Padova Sud IT, Monselice, Italy; 5 Istanbul Kent University, Istanbul, Turkey; 6 Department of CardioVascular Surgery, S. Croce e Carle Hospital, Cuneo, Italy; 7 Department of Cardiovascular Medicine, Heart and Vascular Institute, Cleveland Clinic, Ohio, USA; 8 Department of Internal Medicine, Faculty of Medicine, Cairo University, Cairo, Egypt; 9 Department of Cardiac Surgery, Medical University of Innsbruck, Innsbruck, Austria; 10 The Michael E. DeBakey VA Medical Center, Baylor College of Medicine, Houston, USA

**Keywords:** Cardiac device therapy, Interventional cardiology

## Abstract

Transcatheter aortic valve replacement (TAVR) has shown to reduce mortality compared to surgical aortic valve replacement (sAVR). However, it is unknown which procedure is associated with better post-procedural valvular function. We conducted a meta-analysis of randomized clinical trials that compared TAVR to sAVR for at least 2 years. The primary outcome was post-procedural patient-prosthesis-mismatch (PPM). Secondary outcomes were post-procedural and 2-year: effective orifice area (EOA), paravalvular gradient (PVG) and moderate/severe paravalvular leak (PVL). We identified 6 trials with a total of 7022 participants with severe aortic stenosis. TAVR was associated with 37% (95% CI [0.51–0.78) mean RR reduction of post-procedural PPM, a decrease that was not affected by the surgical risk at inclusion, neither by the transcatheter heart valve system. Postprocedural changes in gradient and EOA were also in favor of TAVR as there was a pooled mean difference decrease of 0.56 (95% CI [0.73–0.38]) in gradient and an increase of 0.47 (95% CI [0.38–0.56]) in EOA. Additionally, self-expandable valves were associated with a higher decrease in gradient than balloon ones (beta = 0.38; 95% CI [0.12–0.64]). However, TAVR was associated with a higher risk of moderate/severe PVL (pooled RR: 9.54, 95% CI [5.53–16.46]). All results were sustainable at 2 years.

## Introduction

Degenerative cardiovascular disease is becoming increasingly prevalent in industrialized countries, due essentially to the aging of the population^1,2^. Aortic stenosis (AS), the most common valvular heart disease in elderly, is associated with high morbidity and mortality^3^. Surgical aortic valve replacement (sAVR) has been the gold-standard method to repair severe AS for decades. However, transcatheter aortic valve replacement (TAVR) has emerged since 2002 as an alternative treatment that has the advantage of being minimally invasive^4^, among several other technical benefits^5^.

The clinical trial journey of TAVR started with the comparison to sAVR in high-risk surgery patients over a decade ago, included intermediate-risk ones some years ago, and ended with low-risk in 2019. All those trials have shown that TAVR is either non-inferior or even superior to sAVR in terms of mortality and other cardiovascular endpoints^6–15^. A first meta-analysis in 2016 regrouping high and intermediate risk patients confirmed those findings and reported a significant 13% decrease in the relative risk of 2-year all-cause mortality in favor of TAVR^16^. A recent update of this metanalysis that included new RCTs of low surgical-risk patients confirmed the benefit in favor of TAVR that was consistent in all surgical risk groups^17^.

Both aortic valvular replacement techniques could be associated with post-operative functional complications. For instance, up to one third of patients experience high post-operative gradients due to a misbalance between the size of the aortic annulus and the orifice area required for an adequate blood perfusion^18^. This condition known as patient-prosthesis mismatch (PPM) is related to diminished regression of the left ventricular mass, bioprosthetic valve dysfunction, symptoms recurrence and unfavorable clinical outcome^19^. There is evidence of increased mortality^20^ and early structural valve deterioration in patients with PPM after aortic valve replacement^21^. Paravalvular regurgitation could also be encountered after valvular replacement. It is related to anatomical irregularities of the calcified tissue and leads to a functional leaking of the valve^22^. Depending on its degree, this kind of regurgitation leads to a volume overload of the left ventricle which secondarily affects the pulmonary circulation and is associated with increased morbidity and mortality after the procedure^23^.

Despite the safety, effectiveness and potential survival benefit of TAVR, there is a gap between the valve performance assessment of this method and clinical outcomes. The aim of this systematic review and meta-analysis is to assess post-procedural echocardiographic parameters in patients with severe AS randomized to TAVR or sAVR.

## Methods

### Literature search

We performed a systematic literature search for randomized controlled trials (RCTs) using 3 databases: Medline, Embase and the Cochrane library, from the 1^st^ of January 2002 till the 20th of December 2019 using specific search terms related to TAVR, sAVR and aortic stenosis/replacement (see supplementary section). The systematic review was performed following the Preferred Reporting Items for Systematic Reviews and Meta-analyses (PRISMA) guidelines^24^ and was registered with the International Prospective Register of Systematic Reviews (PROSPERO identifier: CRD42018115963).

The search was done by 2 independent reviewers (BI, GE) without any language restriction. Any disagreement or inconsistency were resolved by a third reviewer (CAK). References of included trials were further screened for potential inclusion of eligible studies.

### Eligibility criteria

We included RCTs that compared TAVR to sAVR in patients with severe AS, which had a follow-up duration of at least 2 years. Epidemiological data comparing TAVR to sAVR, trials that compared TAVR to any treatment other than sAVR or trials with a shorter follow-up duration were excluded.

### Data extraction

Two independent reviewers extracted data (MK and LS) to a pre-specified data collection sheet. The following information were recorded: trial’s characteristics (name, registration number at clinicaltrials.gov, authors, year of publication) and design (methodology, number of randomized participants, outcome and follow-up duration), patients’ characteristics (age, gender, comorbidities, STS risk score), intervention (prosthesis type, access mode and balloon expansion). Finally, we collected outcome data related to echocardiographic parameters. Any disagreement or inconsistency on recorded data were resolved by another reviewer (NB). All data was extracted at 2 years in the “intention to treat” arms of the trials and was censored beyond that for trials with a longer follow-up period.

### Quality assessment

We assessed the risk of bias in individual RCTs using the revised Cochrane risk of bias tool for randomized trials (RoB 2.0) that measures the risk of bias related to flaws in study design, randomization process, conduct, outcome, analysis and reporting of the data^25^. Overall bias was reported as low risk, some concerns and high-risk.

### Outcomes of interest

The primary outcome was post-procedural patient-prosthesis-mismatch (PPM). PPM was graded according to the indexed effective orifice area (iEOA) after the procedure as follows: absence of PPM: iEOA > 0.85 cm^2^/m^2^ body surface area (BSA), moderate PPM: iEOA between 0.65 and 0.85 cm^2^/m^2^ BSA, severe PPM: iEOA < 0.65 cm^2^/m^2^ BSA. Secondary outcomes were post-procedural and 2-year: effective orifice area (EOA), paravalvular gradient (PVG) and moderate/severe paravalvular leak (PVL).

### Statistical analysis

Forest plots were generated to visualize relative risks (RR) and standardized mean difference (SMD) estimates (for dichotomous and continuous outcomes, respectively) along with their associated 95% CI, for each included RCT, post-procedure, and after 2 years.

Estimates for each outcome were then weighted using the inverse variance method, prior to being pooled using a DerSimonian-Laird random-effects model^26^. This model assumes a normal distribution for true effect sizes (RR or SMD), therefore factoring in the heterogeneity across studies.

Subgroup meta-analyses stratified by patients’ surgical risk on inclusion (high, intermediate and low) or transcatheter heart valve system (balloon and self-expendable) were further performed.

Heterogeneity assessment was conducted by assessing Cochrane’s Q statistic and associated p-value to confirm existence of heterogeneity across studies, and I^2^ to quantify the magnitude of between-study variation that is due to true differences in effect size rather than chance^27,28^.

Univariable meta-regression analysis was also performed to examine and quantify the magnitude of the association between the risk of exposure to echocardiographic parameters post-procedure and at 2 years and patients’ surgical risk and transcatheter heart valve system. RR and β coefficients were calculated along with their 95% CIs. Evidence for an association with risk of exposure to echocardiographic parameters was deemed “strong” at *p* value ≤ 0.05 and “good” at 0.05 < *p* value ≤ 0.1.

Analyses were performed using STATA/SE v15.1 (StataCorp. Stata Statistical Software: Release 15. College Station, TX: StataCorp LP; 2015).

## Results

**Figure 1:**
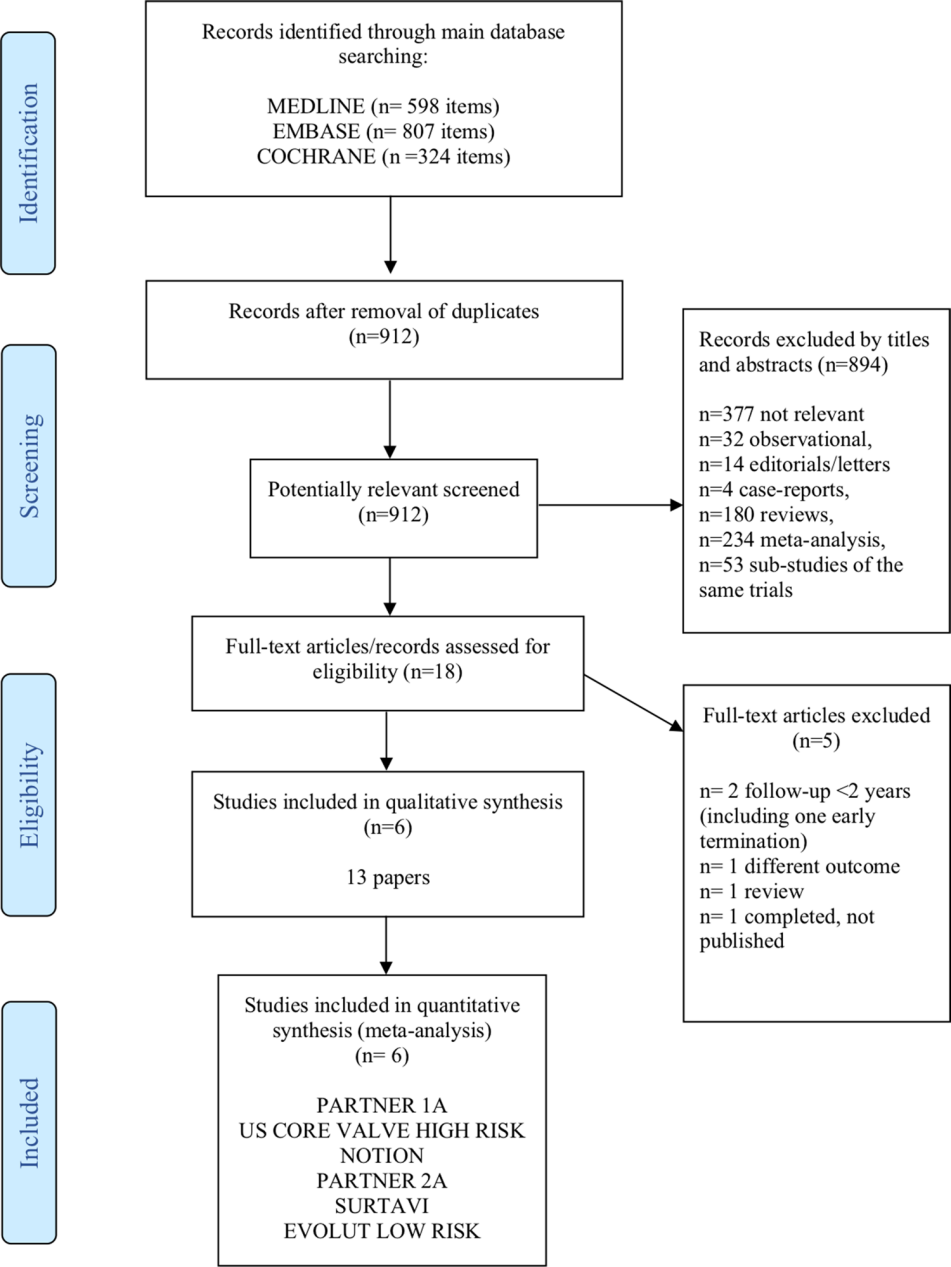
Risk of bias assessment**.**

Our initial search identified 1729 studies from 3 different databases: Medline, Embase and Cochrane. After exclusion of duplicates, 912 studies were screened at the title/abstract level, of which only 18 were deemed to be eligible. Further screening at the full-text level identified 6 RCTs that were included in our meta-analysis (Fig. 1). NOTION^9^, PARTNER 1A^11^, PARTNER 2A^12^, SURTAVI^13^, EVOLUT Low risk^15^ and US CoreValve high risk^29^.

Baseline characteristics of the trials and patients are shown in Table 1. There was a total of 7020 participants, 3511 randomized to TAVR and 3509 randomized to sAVR. Mean age of participants was 80 (3.5) years old, 56.7% of participants were males, almost equally divided in both arms (TAVR arm: mean age is 80.2 (3.4) years old, 56.6% of males; sAVR arm: mean age is 80.4 (3.8) years old, 56.8% are males. All, but NOTION trial, were designed as non-superiority studies. Transfemoral was the most common access route, balloon expandable valves were used in 4 out of the 6 trials. 2 trials included high-risk patients: PARTNER 1A^11^ and US CoreValve high risk^29^, 2 trials included intermediate-risk patients: PARTNER 2A^12^ and SURTAVI^13^; and 2 included low-risk patients: NOTION^9^ and EVOLUT Low risk^15^. All echocardiographic parameters were present except for post-procedural PPM for PARTNER 2A and SURTAVI trials. The risk of bias assessment was overall low (Supplementary table 1).

**Table 1:** Trials and participants characteristics.

	PARTNER 1A		US CoreValve high risk		NOTION		PARTNER 2A		SURTAVI		Evolut Low risk	
	SAVR	TAVI	SAVR	TAVI	SAVR	SAVR	TAVI	SAVR	TAVI	SAVR	SAVR	TAVI
**Trials characteristics**												
ClinicalTrials.gov number	NCT00530894		NCT01240902		NCT01057173		NCT01314313		NCT01586910		NCT02701283	
Number of centers	25		45		3		57		87		86	
Design	Non-inferiority		Non-inferiority		Non-inferiority		Non-inferiority		Non-inferiority		Non-inferiority	
Sample size	348	351	394	401	145	135	1011	1021	879	867	734	734
Recruitment period	2007–2009		2011–2012		2009–2013		2011–2013		2012–2016		2016–2018	
Publication source/year	Kodali et al.^11^		Reardon et al.^29^		Sondergaad et al.^9^		Leon et al.^12^		Reardon et al.^13^		Popma et al.^15^	
Patient’s risk	High		High		Low		Intermediate		Intermediate		Low	
**Participants characteristics**												
Age (years) ± SD	83.6 ± 6.8	84.5 ± 6.4	83.2 ± 7.1	83.5 ± 6.3	79.2 ± 4.9	79.0 ± 4.7	81.5 ± 6.7	81.7 ± 6.7	79.9 ± 6.2	79.8 ± 6.0	74.0 ± 5.9	73.8 ± 6.0
Males (%)	57.8	56.7	53.6	52.9	53.8	52.6	54.2	54.8	57.8	55.8	63.8	66.5
CAD, n (%)	260 (74.9%)	266 (76.9%)	297 (75.4%)	306 (76.3%)	–	–	700 (69.2%)	679 (66.5%)	549 (62.5%)	556 (64.1%)	–	–
Prior cerebrovascular events, n(%)	–	–	51 (12.9%)	53 (13.2%)	24 (16.6%)	22 (16.3%)	–	–	59 (6.7%)	65 (7.5%)	–	–
LVEF, mean (SD)	53 ± 14	53 ± 13	57 ± 13	56 ± 12	57 ± 10	55 ± 10	56 ± 11	55 ± 12	-	-	62 ± 8	62 ± 8
STS* risk score, mean (SD)	11.8 ± 3.3	11.7 ± 3.5	7.3 ± 3.0	7.5 ± 3.2	2.9 ± 1.6	3.1 ± 1.7	5.8 ± 2.1	5.8 ± 1.9	4.4 ± 1.5	4.5 ± 1.6	1.9 ± 0.7	1.9 ± 0.7
**Intervention characteristics**												
Prothesis	Edwards Sapien		Medtronic CoreValve		Medtronic CoreValve		Edwards Sapien XT		Medtronic CoreValve 84% Evolut R 16%		Medtronic CoreValve 3.6% Evolut R 74.1% Evolut Pro 22.3%	
Access route, n (%)												
Transfemoral	244 (70)		294 (100)		145 (100)		775 (77)		503 (100)		731 (99.6)	
Transthoracic	140 (30)		0 (0)		0		236 (23)		0 (0)		3 (0.4)	

### Post-procedural results

**Figure 2:**
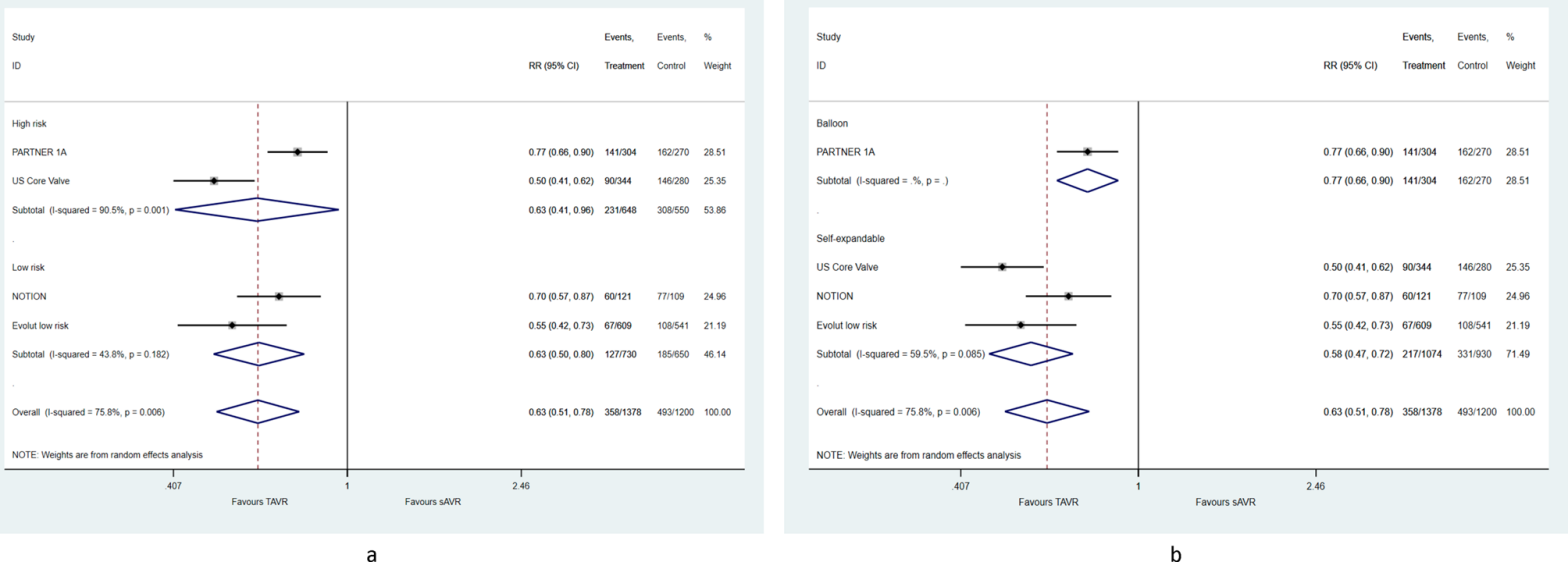
Pooled mean difference of post-procedural patient-prosthesis-mismatch, according to (**a**) surgical risk on inclusion and (**b**) transcatheter heart valve system.

There was a 37% mean relative risk reduction (RR = 0.63, 95% CI [0.51–0.78]) in post-procedural PPM in favor of TAVR. This benefit was observed in high and low surgical risk groups (Fig. 2a), as well as in balloon and self-expendable valves (Fig. 2b) although at different magnitude.

The rest of echocardiographic measures were also in favor of TAVR, except for the PVL.

**Figure 3:**
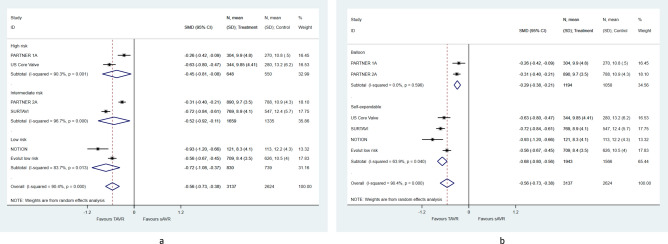
Pooled mean difference of post-procedural gradient at 2 years, according to (**a**) surgical risk on inclusion and (**b**) transcatheter heart valve system**.**

**Figure 4:**
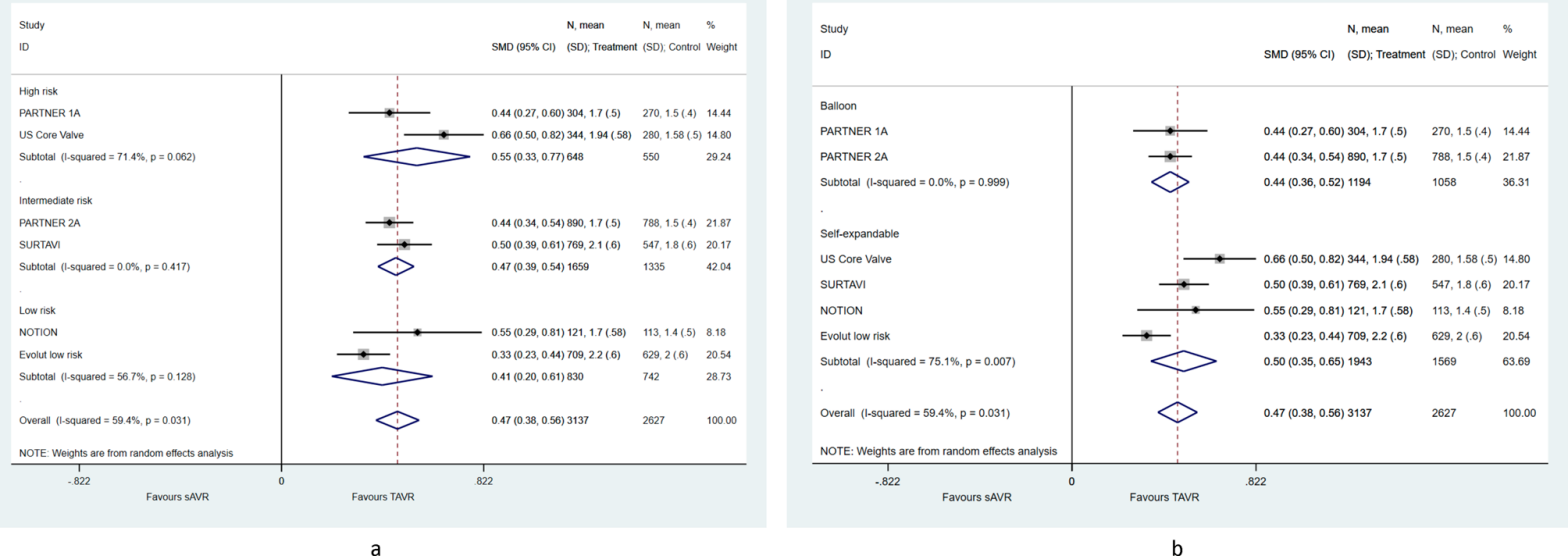
Pooled mean difference of post-procedural effective orifice area at 2 years, according to (**a**) surgical risk on inclusion and (**b**) transcatheter heart valve system.

**Figure 5:**
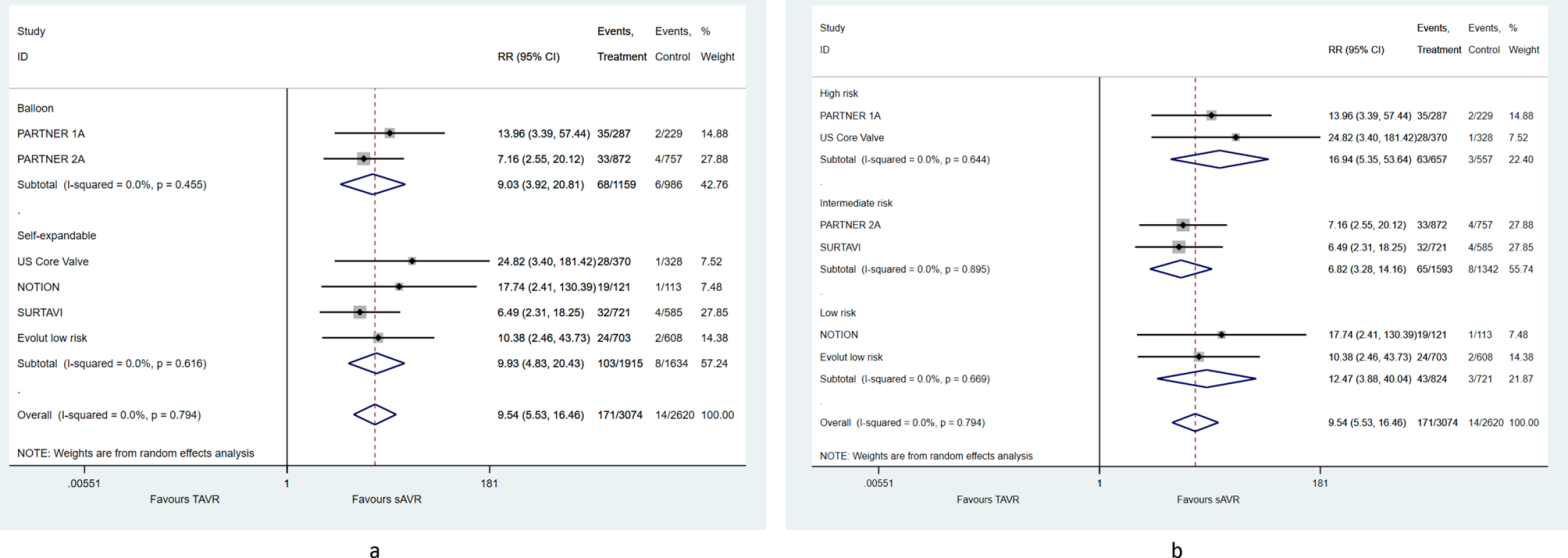
Pooled relative risk of post-procedural moderate/severe paravalvular leak, according to (**a**) transcatheter heart valve system and (**b**) surgical risk on inclusion.

We observed a pooled mean decrease of 0.56 (95%CI [0.73–0.38]) in gradient. Sub-group analysis showed no difference in gradient between TAVR and sAVR across categories of surgical risk on inclusion (Fig. 3a) (*p* = 0.625). However, self-expendable valves were associated with a larger decrease in gradient than balloon ones (Fig. 3b) (β = − 0.38; 95% CI [− 0.64, − 0.12]). We also observed an overall increase of 0.47 (95% CI [0.38–0.56]) in EOA. However, the postoperative EOA did not differ between self-expandable and balloon expandable valves. The latter was consistent across subgroups (Fig. 4a,b). Finally, TAVR was associated with an almost tenfold increase in the risk of moderate/severe PVL (pooled RR: 9.54, 95% CI [5.53–16.46]), that was noticed in both subgroups (Fig. 5a,b).

### 2-year outcome

A similar trend was observed at 2 years. We noted a pooled mean decrease of 0.59 (95%CI [0.29–0.89]) in gradient that was independent of the patient’s surgical risk at inclusion (Supplementary Fig. 1a). However, self-expandable valves were associated with a larger gradient decrease as compared to balloon-expandable ones (β = − 0.62; 95%CI [− 0.85, − 0.40]) (Supplementary Fig. 1^1^). Additionally, there was a pooled mean increase of 0.46 (95% CI [0.25–0.67]) in EOA that was significant in all surgical risk categories (Supplementary Fig. 2a). However, self-expendable-valves were associated with a larger increase in EOA compared to balloon ones (β = 0.35; 95% CI [0.01–0.70]) (Supplementary Fig. 2b). Finally, the risk of moderate/severe PVL was almost tenfold higher in patients who had a valvular replacement with TAVR two years earlier (pooled RR: 10.20, 95%CI [4.84–21.49]). Those findings were consistent in both-groups (Supplementary Figs. 3a and 3b and 4b).

## Discussion

This meta-analysis focused on quantitative and qualitative echocardiographic outcomes reviewing RCTs that comparing TAVR and sAVR for the treatment of severe AS. The higher effective orifice areas of the aortic prosthesis and the lower residual gradients after TAVR speak for a more effective treatment of the disease by TAVR as compared to sAVR. The lower rates of PPM after TAVR also support the higher effectiveness of treatment by TAVR. These findings are certainly counterbalanced by the significantly lower rates of paravalvular regurgitation in sAVR patients.

The major strength of the study is the acquisition of echocardiographic parameters with other hard clinical endpoints such as all-cause mortality. Studying echocardiographic parameters closes the gap of shortness of follow-up of several aortic stenosis RCTs. Those parameters are supposed to be the best predictors of mortality and morbidity after the treatment of AS. The echocardiographic results obtained by the meta-analysis may explain some of the differences between the two treatment arms in terms of mortality: TAVR has the advantage of low residual gradients (and lower rates of PPM) but the disadvantage of higher rates of paravalvular regurgitation. As both conditions can be associated with increased mortality, any improvement on the incidence of PVL in TAVR valve or PPM in surgical valve may let the cards be reshuffled again.

The studies included in the analysis refer to the use of SAPIEN and SAPIEN XT (represent the balloon expandable valves family) and Corevalve and Evolute R (self-expandable valves). A head-to-head comparison of the PARTNER 1, 2 and 3 trials demonstrate a gradual reduction of the rates of relevant paravalvular regurgitation (greater than or equal to moderate) from 12.2, to 3.4%, to 0.8%, respectively. Mild paravalvular regurgitation was detected in 65.2%, to 20.4%, to 28.7% respectively. The impact of paravalvular regurgitation on mortality was significant in patients with PVL greater than or equal to mild in the PARTNER I trial and PVL greater than moderate in PARTNER 2. This discrepancy is most probably related to the fact that the numbers of patients with paravalvular regurgitation dropped overtime and the PARTNER 2 trial was underpowered to address the effect of mild PV-leak in those patients. As demonstrated by the results, the numbers of PV leaks greater than trace significantly change between the SAPIEN and SAPIEN XT prosthesis, but not between SAPIEN XT and SAPIEN 3 in those randomized studies. The same trend is observed for self-expandable valves too. Taken together, the results indicate a trend for decreased paravalvular regurgitation greater than or equal to moderate, but the overall occurrence of the finding remained unchanged. There are some observational studies demonstrating improved results in the newest generation of transcatheter valves, however their real impact on hard clinical outcomes is still unknown^30^.

Interestingly we demonstrated that there are differences in EOA, transvalvular gradients and PPM not only between the two treatment arms but also within the TAVR valves. Self-expandable valves have been found to be more advantageous than balloon-expandable valves. This is also supported by registry data in the literature^31^. However, literature data demonstrate a higher incidence of PVL in self-expandable valves, though this has not been investigated in our analysis^32^. From this point of view, our meta-analysis provides first evidence that patients at risk for PPM may benefit from transcatheter treatment especially by using a self-expandable valve. On the other hand, patients at risk for PVL should be rather treated by conventional surgery. From a different angle, our results are completely aligned with a recent meta-analysis that compared only PPM in both procedures and reported a benefit towards TAVR irrespective of the study design, severity of the disease and follow-up period^33^. All randomized trials included into the meta-analysis were related to the surgical risk assessed by common surgical risk scores. The most important lesson from those trials is that the surgical risk may affect the perioperative and mid-term survival of sAVR as well as the mid-term survival of TAVR patients but not the perioperative survival of the latter. The anatomical risk on the degree and distribution of calcification as well as the presence of a bicuspid valve or left ventricular outflow tract (LVOT) calcifications has been associated with inferior results in TAVR^34–36^. This knowledge became more evident during the evolution of TAVR and patients with high anatomical risk conditions have been excluded from randomization. Still the surgical risk is very well depicted in patients’ selection of the different trials and has been separately analyzed in Figs. 2, 3, 4 and 5. As expected, the surgical risk did not affect residual gradients, the effective orifice area after treatment, nor the rates of paravalvular regurgitation.

Our results at 2 years confirmed the immediate postoperative results and indicate that the margin of changes both in terms of EOA and residual gradients, as well as PVL is very small at mid-term. Whether those differences reflect the long-term echo findings and affect valve function in the long run is still unknown. The presence of the calcified aortic valve tissue near the bioprosthetic valve, the crimping manipulation and the non-circular expansion of the transcatheter valve prosthesis may turn the scales towards conventional surgical prostheses^37^. Up to now, the effect of crimping on the pericardial tissue was not associated with any clinical disadvantage. However, there are laboratory trials demonstrating irreversible tissue damage on pericardial leaflets especially in aggressive crimping manoeuvres (< 16 French)^38,39^. On the other hand, the presence of a surgical sutures and Teflon pledges^40^ in the left ventricular outflow tract and the crown-shaped design of a common surgical bioprosthesis may increase turbulences within the heart cycle and promote thrombogenicity^41^. The latter is a known factor of early valve dysfunction and degeneration.

We acknowledge the presence of some limitations in our study. Although the overall risk of bias was low, there are still some possibilities of outcome measurement bias in the studies, especially for the measurement of echocardiographic parameters that are operator- and technique- dependent. As pointed out by the subgroup analysis, the type of prosthesis may also play a role at the high degree of heterogeneity of the echocardiography results. The use of different types of prosthesis was only investigated within the TAVR arm, due to the lack of data at the surgical arm. Although surgical prostheses do not variate a lot, some degree of heterogeneity on the grounds of prostheses differences cannot be excluded. All studies included different models of the same prosthesis including also early generation devices. Newer TAVR prostheses are associated with lower rates of paravalvular leak, whereas newer surgical prostheses are related to improved EOA and residual gradients. To which extent this variability in both treatment arms has influenced all types of outcomes presented in the meta-analysis remains unknown. The eligibility criteria for recruitment in the studies included were based on risk stratification. The latter was performed by using scores which were basically developed for surgical patients. The use of the STS PROM score is widely accepted -mainly due to the lack of alternatives-, however this score may not accurately reflect the perioperative risk after TAVR. The inclusion criteria for eligible participants in prospective randomized trials are carefully selected and may not always reflect daily practice^42^. However, the majority of data from audited national and multicenter registries mostly confirm the presented results. Finally, with only 6 trials included in our meta-analysis, it was not possible to perform a meta-regression that takes into account confounding factors like age, gender and cardiovascular risk factors.

## Conclusion

The effective orifice area, the transvalvular gradients and the patient-prosthesis mismatch favor transcatheter aortic valve replacement over surgery for the treatment of severe aortic stenosis in our metanalysis. This benefit is counterbalanced by higher rates of paravalvular regurgitation. Nevertheless, the effect of newer generation prostheses both in transcatheter and in surgical aortic valve replacement still needs to be determined. Future research should focus on the effect of these echocardiographic differences on clinical outcomes.

## Supplementary Information

Supplementary Information.

## Data Availability

The data that support the findings of this study are available from the authors upon reasonable request from the corresponding author.
