# Supplementary material for: Aortic valve function post-replacement of severe aortic stenosis by transcatheter procedure versus surgery: a systematic review and metanalysis
Source: Sci Rep. 2021 Jun 7;11:11975. doi: 10.1038/s41598-021-91548-x (PMC8184892; doi:10.1038/s41598-021-91548-x)

**Aortic valve function post-replacement of severe aortic stenosis by transcatheter procedure versus surgery: A systematic review and metanalysis.**

Charbel Abi Khalil, MD, PhD ^1,2,3^, Barbara Ignatiuk, MD ^3,4 *^, Guliz Erdem, MD ^3,5 *^, Hiam Chemaitelly, MSc ^1^, Fabio Barilli, MD ^6^, Mohamed El-Shazly MD ^1,7^, Jassim Al Suwaidi, MBChB ^1^, Samar Aboulsoud, MBChB ^8^, Markus Kofler, MD ^8^, Lukas Stastny, MD ^9^, Hani Jneid, MD ^10^ **, Nikolaos Bonaros, MD ^3,9^ **

* Equal collaboration

** Co-last authors

1. Research Department. Weill Cornell Medicine-Qatar. Doha, Qatar.
2. Joan and Sanford I. Weill Department of Medicine. Weill Cornell Medicine. New York, US
3. London School of Economics, Department of Health Policy. London, UK
4. Ospedali Riuniti Padova Sud IT. Monselice, Italy
5. Turkey Istanbul Kent University. Istanbul,Turkey
6. Department of CardioVascular Surgery. S.Croce e Carle Hospital, Cuneo, Italy
7. Department of Cardiovascular Medicine, Heart and Vascular Institute, Cleveland Clinic. Ohio, US.
8. Faculty of Medicine. Cairo university. Egypt, Cairo
9. Department of Cardiac Surgery, Medical University of Innsbruck. Innsbruck, Austria.
10. The Michael E. DeBakey VA Medical Center. Baylor College of Medicine. Houston, US

**Supplementary materials**

**Supplementary section:** Search algorithms:

**MEDLINE**

#1. Transcatheter Aortic Valve Replacement/ 4747

#2. (trans?catheter or percutaneous or trans?cutaneous or trans?arterial or

trans?vascular or trans?femoral or trans?apical or trans?axillar* or trans?aortic or

trans?subclavian or trans?carotid or TAVI or TAVR).mp. [mp=ti, ab, hw, tn, ot,

dm, mf, dv, kw, fx, dq, nm, kf, ox, px, rx, ui, sy] 193973
#3. 1 or 2 193973
#4. exp Aortic Valve Stenosis/ 40782
#5. "aortic stenosis".mp. 17505
#6. 4 or 5 45675
#7. (replacement or implantation).mp. [mp=ti, ab, hw, tn, ot, dm, mf, dv, kw, fx,

dq, nm, kf, ox, px, rx, ui, sy] 511390

#8. random*.tw. 1101085
#9. 3 and 6 and 7 and 8 604

#10. limit 9 to yr =”2002-2019” 589

**EMBASE**

#1. Transcatheter Aortic Valve Replacement/ 18567

#2. (trans?catheter or percutaneous or trans?cutaneous or trans?arterial or

trans?vascular or trans?femoral or trans?apical or trans?axillar* or trans?aortic or

trans?subclavian or trans?carotid or TAVI or TAVR).mp. [mp=ti, ab, hw, tn, ot,

dm, mf, dv, kw, fx, dq, nm, kf, ox, px, rx, ui, sy] 340337

#3. 1 or 2 340337
#4. exp Aortic Valve Stenosis/ 2716 29041

#5. "aortic stenosis".mp. 29041
#6. 4 or 5 30672
#7. (replacement or implantation).mp. [mp=ti, ab, hw, tn, ot, dm, mf, dv, kw, fx,

dq, nm, kf, ox, px, rx, ui, sy] 669588

#8. random*.tw. 1494029
#9. 3 and 6 and 7 and 8 807

#10. limit 9 to yr =”2002-2019”

**COCHRANE**

#1 MeSH descriptor: [Aortic Valve Stenosis] this term only 600

#2 aortic near stenos?s 1417

#3 #1 or #2 1417

#4 transcatheter aortic valve implantation 758

#5 MeSH descriptor: [Transcatheter Aortic Valve Replacement] explode all trees 138

#6 #4 or #5 809

#7 #3 and #6 525

#8 #7 and surg* 443

#9 #8 and randomi*

with Publication Year from 2002 to 2019, in Trials 324

**Supplementary table 1:** Risk of bias assessment using the Rob 2.0 tool.

|  | **PARTNER 1A** | **US CoreValve**  **High Risk** | **NOTION** | **PARTNER 2** | **SUR-TAVI** | **EVOLUT LOW RISK** |
| --- | --- | --- | --- | --- | --- | --- |
| **Study design** | Randomized open label, parallel  group | Randomized, open label, parallel  group | Randomized, open label, parallel  group | Randomized, open label, parallel  group | Randomized,  open label, parallel  group | Randomized, open label, parallel group |
| **Outcome assessed** | Death from any cause | Rate of death from any cause | Combined outcome measure consisting of death from any cause, myocardial infarction, and stroke | Death from any cause or disabling stroke | All-cause mortality or disabling stroke | Composite of death from any cause or disabling stroke at 24 months |
| **Aim** | to determine the safety and effectiveness of the device and delivery systems (transfemoral and transapical) in high surgical risk patients with severe AS | to assess the safety and effectiveness of TAVI with a self-expanding prosthesis as compared with SAVR in patients with severe AS | to compare TAVI using Core Valve System and SAVR in patients with severe AS  in a cohort of low-risk, moderate risk and high-risk patients | to assess the safety and efficacy of balloon expandable TAVR compared with SAVR in AS patients with intermediate surgical risk | To compare the safety and efficacy of TAVR performed with the use of self-expanding bioprothesis with SAVR in AS patients with intermediate surgical risk | to evaluate the safety and effectiveness of TAVR with a self –expanding bioprosthesis compared with SAVR in AS patients with low surgical risk. |
| **Sources** | Journal articles and trial  protocol | Journal articles and trial protocol | Journal articles and trial protocol | Journal articles and trial protocol | Journal articles and trial protocol | Journal articles and trial protocol |
| **Bias arising from the randomization process** | | | | | | |
| **1.1** | Yes | Yes | Yes | Yes | Yes | Yes |
| **1.2** | Yes | NI | PY | PY | Yes | PY |
| **1.3** | No | No | No | No | No | No |
| **Risk of bias** | Low | Low | Low | Low | Low | Low |
|  |  |  |  |  |  |  |
| **Bias due to deviations from intended interventions** | | | | | | |
| **2.1** | Yes | Yes | Yes | Yes | Yes | Yes |
| **2.2** | Yes | Yes | Yes | Yes | Yes | Yes |
| **2.3** | Yes | No | No | PN | No | No |
| **2.4** | No | - | - | - | - | - |
| **2.5** | No | No | No | No | PY | No |
| **2.6** | - | - | - | - | No | - |
| **Risk of bias** | Low | Low | Low | Low | Low | Low |
| **Bias due to missing outcome data** | | | | | | |
| **3.1** | PY | PY | PY | PN | PN | PN |
| **3.2** | - | - | - | No | PN | PY |
| **3.3** | Yes | Yes | Yes | Yes | Yes | Yes |
| **Risk of bias** | Low | Low | Low | Low | Low | Low |
| **Bias in measurement of the outcome** | | | | | | |
| **4.1** | No | PN | PN | PN | PN | No |
| **4.2** | - | - | - | - | - | - |
| **Risk of bias** | Low | Low | Low | Low | Low | Low |
| **Bias due to selection of the reported result** | | | | | | |
| **5.1** | No | No | No | No | No | No |
| **5.2** | No | No | No | No | No | No |
| **Risk of bias** | Low | Low | Low | Low | Low | Low |
|  |  |  |  |  |  |  |
| **Overall Bias** | **Low** | **Low** | **Low** | **Low** | **Low** | **Low** |

**Supplementary Figure 1:** Pooled mean difference of gradient at 2 years, according to (a) surgical risk on inclusion and (b) transcatheter heart valve system


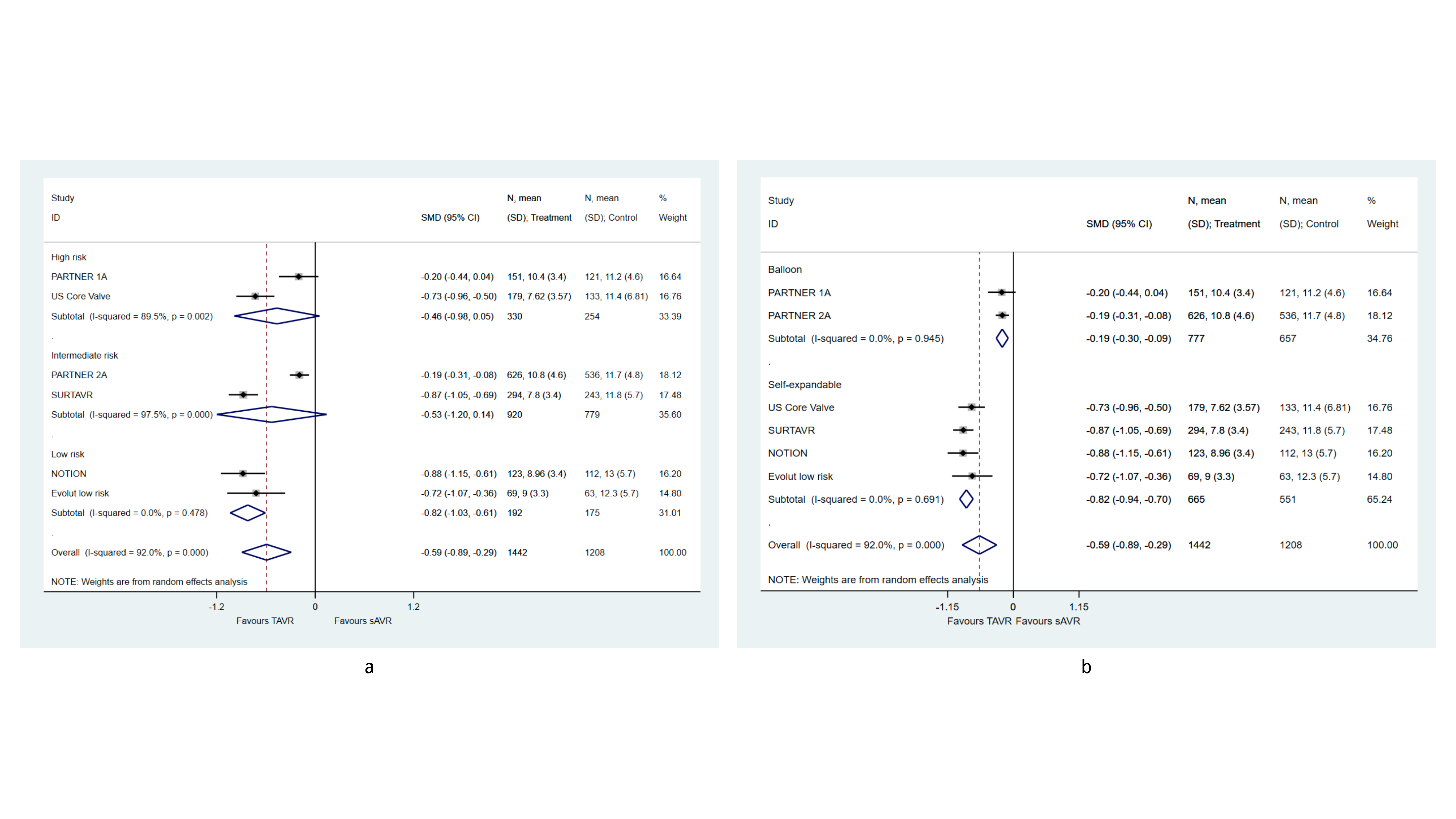


**Supplementary Figure 2:** Pooled mean difference of effective orifice area at 2 years, according to (a) surgical risk on inclusion and (b) transcatheter heart valve system


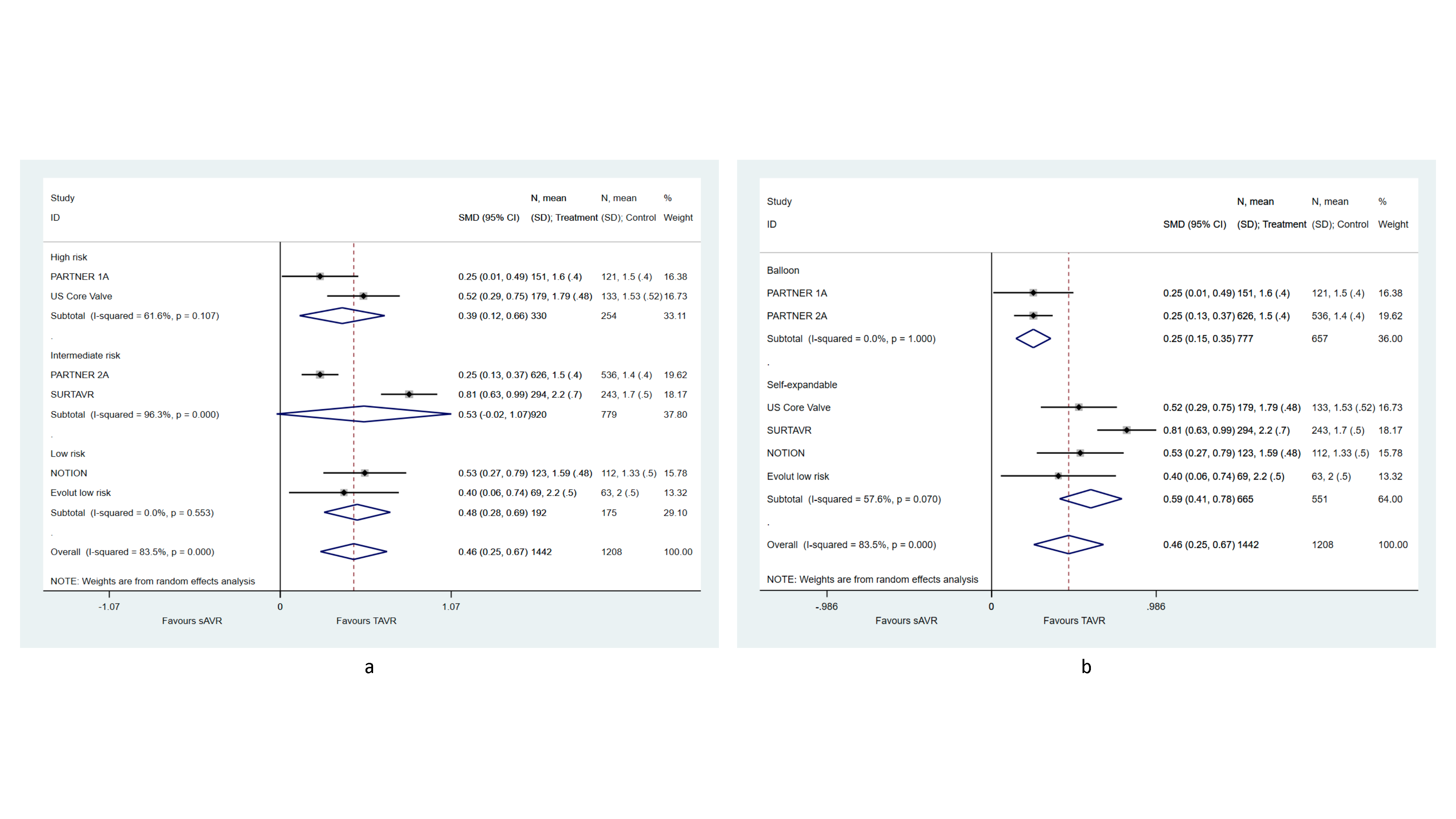


Supplementary Figure 3**:** Pooled relative risk of moderate/severe paravalvular leak at 2 years, according to (a) surgical risk on inclusion and (b) transcatheter heart valve system


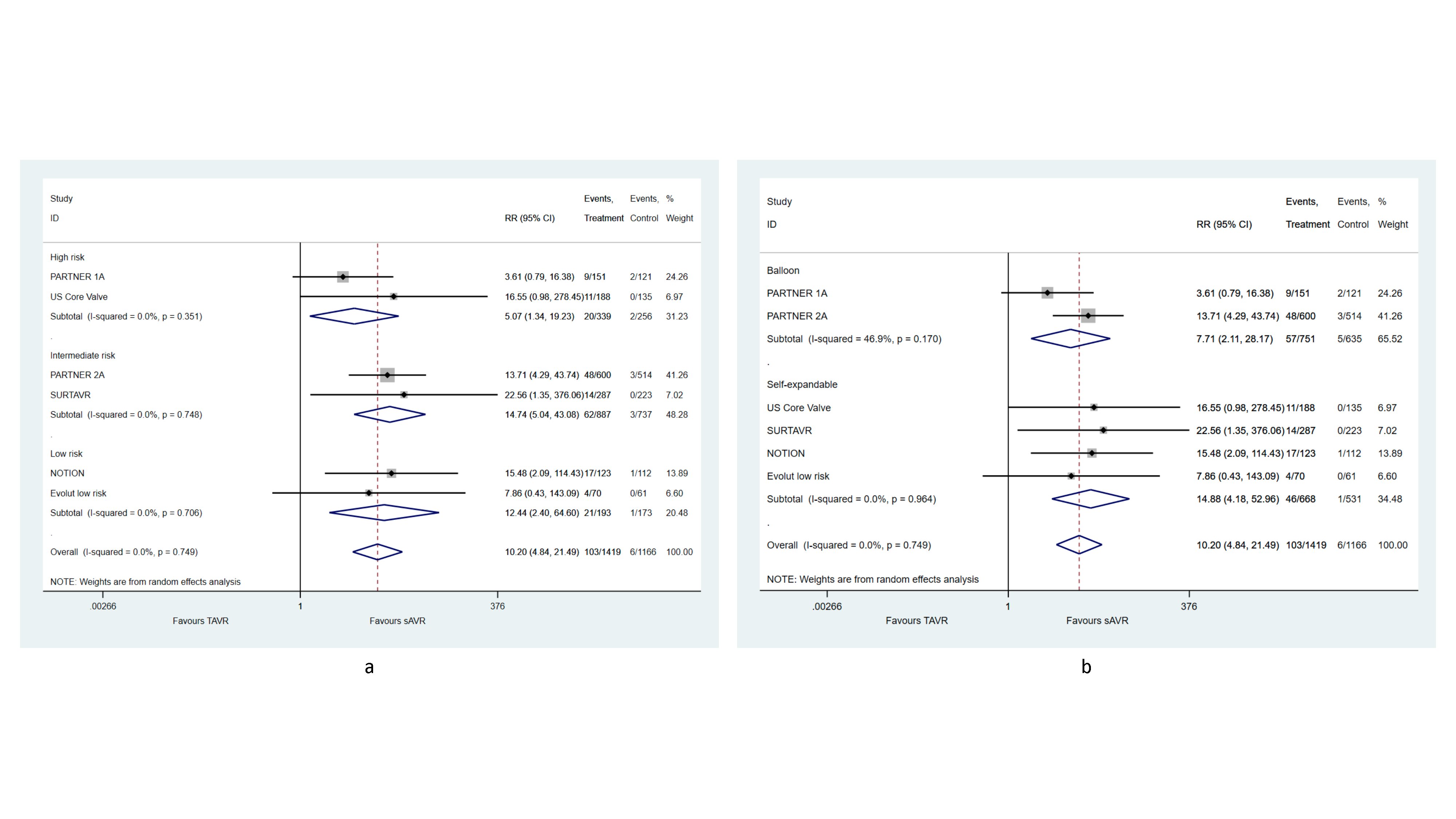

Supplement: Supplementary file 1 — Supplementary Information. [file 41598_2021_91548_MOESM1_ESM.docx]
